# Inflammation, Endothelial Dysfunction and Increased Left Ventricular Mass in Chronic Kidney Disease (CKD) Patients: A Longitudinal Study

**DOI:** 10.1371/journal.pone.0138461

**Published:** 2015-09-23

**Authors:** Kyriakos Ioannou, Vianda S. Stel, Evangelia Dounousi, Kitty J. Jager, Aikaterini Papagianni, Konstantinos Pappas, Kostas C. Siamopoulos, Carmine Zoccali, Dimitrios Tsakiris

**Affiliations:** 1 Nephrology Department, Nicosia General Hospital, Nicosia, Cyprus; 2 ERA–EDTA Registry, Department of Medical Informatics, Academic Medical Center, University of Amsterdam, Amsterdam, The Netherlands; 3 Department of Nephrology, Medical School, University of Ioannina, Ioannina, Greece; 4 Department of Nephrology, Aristotle University of Thessaloniki, Hippokration General Hospital, Thessaloniki, Greece; 5 Department of Cardiology, University Hospital of Ioannina, Ioannina, Greece; 6 CNR–IBIM Clinical Epidemiology and Pathophysiology of Renal Diseases and Hypertension, Renal and Transplantation Unit, Reggio Calabria, Italy; 7 Department of Nephrology, Papageorgiou General Hospital of Thessaloniki, Thessaloniki, Greece; Fondazione G. Monasterio, ITALY

## Abstract

**Introduction:**

Within this longitudinal study we investigated the association of inflammation markers C-reactive protein (CRP), interleukin-6 (IL-6) and tumor necrosis factor-α (TNFα) and endothelial dysfunction markers intercellular adhesion molecule-1 (ICAM-1) and vascular cell adhesion molecule-1 (VCAM-1) with left ventricular mass indexed for height^2^·^71^ (LVMI) in hypertensive predialysis CKD patients.

**Material and Methods:**

From 2004 to 2005, 182 incident consecutive adult patients from the outpatient CKD clinics of two hospitals in Greece with CKD and hypertension or using antihypertensive medication, were included. Of these, 107 patients underwent CRP (mg/l) and LVMI (g/height^2^·^71^) measurements annually for three years.

**Results:**

In the longitudinal analyses, using linear mixed modeling, a higher IL-6 (ß = 1.9 (95%ci:0.38;3.5), inflammation score based on CRP, IL-6 and TNF-α (ß = 5.0 (95%ci:0.72; 9.4) and VCAM-1 (ß = 0.01 (95%ci:0.005;0.02) were associated with higher LVMI. These models were adjusted for age, gender and primary renal disease, and for confounders that on top changed the beta with ≥10%, i.e. diuretic use (for IL-6 and inflammation score).

**Conclusion:**

The results suggest that in predialysis CKD patients, inflammation as well as endothelial dysfunction may play an important role towards the increase in LVMI.

## Introduction

Left ventricular hypertrophy (LVH) is perhaps the strongest integrator of cardiovascular risk in hypertensive patients [1]. Moreover, LVH is an exceedingly common co-morbidity in chronic kidney disease (CKD) patients whose prevalence is strictly related to the severity of CKD [2]. Reduction of left ventricular mass (LVM) signals a reduction in the risk of cardiovascular events and mortality [3] both in hypertensive patients [1] and in patients with stage 5-CKD on dialysis [4]. Although hypertension, volume overload and anemia are considered as dominant risk factors for LVH in CKD, these factors only in small part explain the variability in LVM in CKD [2]. Thus the identification of novel risk factors for LVH in CKD appears to be of paramount importance if we are to curb the high mortality risk associated with this alteration in CKD patients.

Both inflammation and endothelial dysfunction markers are potential risk factors for LVH in CKD patients. Experimental models and a variety of clinical studies in systemic hypertension and in heart failure document that inflammation is fundamental for the development and progression of myocardial hypertrophy and for the evolution of this alteration towards overt heart failure [5]. Moreover, the prevalence of inflammation is quite high both in CKD patients prior to initiation of dialysis [6–8] and in dialysis patients [9–11], and in these patients, inflammation appeared to be a potential risk factor for cardiovascular morbidity and mortality [11–15]. Also, in salt overloaded spontaneously hypertensive rats LVH and endothelial dysfunction are strictly inter-related phenomena [16] and both these alterations are hallmarks of salt-sensitive hypertension in humans [17]. However, so far, the number of studies investigating the association of inflammation or endothelial dysfunction markers with LVM is very limited in pre-dialysis CKD patients [18–21], especially across the whole spectrum of predialysis CKD, and these studies all had a purely cross-sectional design.

With this background in mind we designed a longitudinal study aimed to investigate the association of inflammation markers C-reactive protein (CRP), interleukin-6 (IL-6) and tumor necrosis factor-α (TNFα) and endothelial dysfunction markers intercellular adhesion molecule-1 (ICAM-1) and vascular cell adhesion molecule-1 (VCAM-1) with LVM indexed for height^2^·^71^ (LVMI) in predialysis CKD patients with hypertension or using antihypertensive medication.

## Materials and Methods

### Patients

This study includes consecutive incident adult CKD patients who visited the outpatient CKD clinics of two hospitals in Greece in 2004 or 2005. Prior to the study, patients had been followed up for three months to confirm the presence of CKD. The definition of CKD was based on the guidelines of the Kidney Disease Outcomes Quality Initiative [22], while the management of CKD patients was based on the various guidelines/ suggestions [22–25]. Exclusion criteria were [1] a history of malignancy after which patients were free of disease for less than five years, and [2] the presence of clinical infection or [3] the presence of a major cardiovascular event, defined as stroke, myocardial infarction or acute ischemic heart disease, during the last three months prior to study entry.

In total, 206 CKD patients fulfilled the criteria as described above. Of these, we excluded 24 CKD patients with (1) no hypertension (getting a more homogeneous patient population as hypertension is an important risk factor for increased LVM); (2) no baseline data on the independent variables (inflammation markers and endothelial dysfunction markers); or (3) no baseline data on the outcome variable (LVM). As such, this study included 182 CKD patients (= 88% of total study population). Data is provided in S1 File.

Hypertension was defined as systolic blood pressure ≥ 140 mmHg, diastolic blood pressure ≥ 90 mmHg or the use of antihypertensive medication. The study was approved by the Ethical Committee of the General Hospital of Veria and the Ethical Committee of the University Hospital of Ioannina, and patients participated after providing written informed consent.

### Data collection at baseline and follow-up

At study entry the patients underwent a detailed review of their medical history and careful clinical examination. Additionally, baseline characteristics, including demographics, primary renal disease, diabetic status, smoking habits, medication, and blood pressure were assessed. A full haematology and biochemical screen was performed, whereas estimated glomerular filtration rate (eGFR) (ml/min/1.73m²) was calculated by the Chronic Kidney Disease Epidemiology Collaboration (CKD-EPI) equation [26]. Additionally, LVM was assessed annually for three years in 107 patients (of which 64% had all three annually follow-up measurements of LVM). In these 107 patients, serum CRP was also annually assessed for three years (of which 80% had all three annually follow-up measurements of CRP).

### Laboratory measurements

Blood samples were taken from a peripheral vein under fasting conditions. Serum samples were separated from clotted blood (after 30 min) by centrifugation (10 to 15 min at approximately 1000 x g), aliquoted and stored at -80°C until assay. Samples were measured between 2006 and 2007, at the Laboratory for Study of Kidney Diseases of the Department of Nephrology, Aristotle University of Thessaloniki, Hippokration Hospital of Thessaloniki. For this storage period, samples are considered quite stable and the degradation rate low (variation < 15%). Samples aliquots were thawed just once for the measurement. Serum levels of ICAM-1 and VCAM-1 were measured by a sandwich enzyme immunoassay technique (ELISA) using commercially available standard kits (Quantikine human sICAM-1 and human VCAM-1, Research & Diagnostic Systems Europe Ltd, Abington UK). ELISA system sensitivity was <2 ng/ml for both ICAM-1 and VCAM-1. Serum IL-6 and TNF- α were measured by a high sensitivity ELISA (Quantikine HS human IL-6 and TNF- α, Research & Diagnostic Systems Europe Ltd, Abington UK). Sensitivity of the ELISA system was <0.5 pg/ml for both IL-6 and TNF-α. The intra-assay coefficient of variation (CV%) was 3.5 to 6.8% for ICAM-1 and VCAM-1 and 7.1 to 10.3% for IL-6 and TNF-α. The inter-assay CV% was 6.2 to 9.4% for ICAM-1 and VCAM-1 and 7.3 to 10.8% for IL-6 and TNF-α. The concentrations of all molecules were calculated by reference to standard curves performed with the corresponding recombinant molecule. All serum samples were tested in duplicate. In 30 healthy blood donor volunteers ICAM-1 values ranged from 157–480 ng/ml, VCAM-1 327–750 ng/ml, IL-6 0.552–7.18 pg/ml and TNF-α <1.0 pg/ml (data not shown).

Serum CRP levels were measured by high sensitivity immunoturbidimetry, enhanced latex technology (Cobas Integra 800, Roche). The lower detection level was 0.1mg/L and measuring range was 0.1–306 mg/L. Intermediate precision was for a mean CRP level 13.3 mg/L (CV: 2.1%) and for mean CRP level 0.53 mg/L (CV: 8.4%). Expected range for adults is <5.0 mg/L. Cut-off points for CVD risk assessment: <1.0 mg/L low, 1.0–3.0 mg/L average, >3.0 mg/L high [27].

### Measurement of LVM

LVM was assessed by 2D-mode echocardiogram, by a single cardiologist from each hospital who followed a predefined protocol as is described more extensively elsewhere [28].

### Outcome variables

The primary outcome was LVM indexed for height (g/m^2^·^71^) (LVMI). The height-based indexing of LVM was specifically chosen to minimize any potential distortion attributable to extracellular volume expansion (surface area indexing being weight-sensitive), as recommended in previous studies in CKD [2, 29].

### Inflammation score

In accordance to a previous study [30], we calculated an inflammation score based on CRP, IL-6 and TNF-α. First, the highest tertile of the corresponding variable distribution was calculated [29] resulting in corresponding cut-off points for CRP (>4 mg/l), IL-6 (>4 pg/ml) and TNF-α (>2.5 pg/ml). Thereafter, the inflammation score was calculated having 0 points (if CRP, IL-6 and TNF-α were *not* in the highest tertile), 1 point (if CRP, IL-6 *or* TNF-α was in the highest tertile), 2 points (if CRP and IL-6, CRP and TNF-α, or IL-6 and TNF-α were in the highest tertile) or three points (if CRP, IL-6 *and* TNF-α were in the highest tertile).

### Statistical analysis

To compare the patient characteristics across LVMI categories (in tertiles) we used tests for trend of means (for normally distributed data), and medians (for non-normally distributed data), and a chi-square test for trend provided (for categorical variables). A two-tailed p-value lower than 0.05 was considered as statistically significant.

For the cross sectional analysis, we used linear regression analysis to test the association between both inflammation markers and endothelial dysfunction markers with LVM. Model 1 was an unadjusted model. Model 2 was adjusted for three main variables fulfilling the criteria for confounding [31] i.e. age, sex, and primary renal disease plus confounders that, on top, changed the beta with ≥10%, i.e. smoking, ace or arb use, diuretic use and statin use (log CRP); tryglycerides and diuretic use (IL-6); smoking (log TNF-α); and diuretic use (inflammation score). So the models with the independent variables of ICAM-1 and VCAM –I were adjusted for age, gender and primary renal disease only. Model 3 was adjusted for the confounders of model 2 plus eGFR, which did not qualify the criteria for confounding but was expected to be a mediator [31] and was adjusted for, in order to examine to what extent the associations were mediated by eGFR.

For the longitudinal analyses, linear mixed models were used to analyze the association between the inflammation markers and endothelial dysfunction markers with LVM. Linear mixed models appeared to be most suitable for the analysis of longitudinal data, and provide effect estimates unaffected by drop out of follow-up measurements [32]. Moreover, linear mixed modeling can be used if the independent variable is measured annually over time (CRP) or at baseline only (Il-6, TNF-α, ICAM-1 and VCAM-1) [32]. For these longitudinal analyses we performed the same three models as described above., We compared the beta’s of log CRP and their accompanying confidence intervals of the fully adjusted model (model 2) with models including less confounders (in different combinations), both in the cross sectional and longitudinal analyses. In our analyses we did not adjust for body weight, because our primary outcome LVMI was indexed for body surface area. The analyses were performed with IBM SPSS Statistics 19 and SAS version 9.2.

## Results

Table 1 shows the characteristics for the study population at baseline (n = 182) and for those with at least one follow-up measurement on LVM (n = 107), overall and by LVMI tertile. At baseline, the median (25^th^-75^th^ percentile) age was 68.1 (60.5–73.4) years, median CRP was 2.0 (0.73–4.5) mg/l, median IL-6 was 3.1 (2.1–4.5) pg/ml, median TNF-α was 1.9 (1.4–2.9) pg/ml, median ICAM-1 was 254.5 (205.5–328.4) ng/ml, and median VCAM-1 was 842.3 (623.0–1148.3) ng/ml. Furthermore, the median baseline LVM indexed for height was 69.4 g/m^2^·^71^ (males 70.6 g/m^2^·^71^; females 67.3 g/m^2^·^71^).

**Table 1:** Baseline characteristics in chronic kidney disease patients with hypertension, by height indexed left ventricular mass (LVMI) in tertiles.

	Patients with baseline data on left ventricular mass (n = 182)					Patients with follow-up data on left ventricular mass (n = 107)				
	Baseline LVMI (g/m^2^·^71^)					Baseline LVMI (g/m^2^·^71^)				
	All	Tertile 1 <55	Tertile 2 ≥55 and <75	Tertile 3 ≥75	P values, test for trend	All	Tertile 1 <55	Tertile 2 ≥55 and <75	Tertile 3 ≥75	P values, test for trend
Number	182	46	67	69		107	28	37	42	
Age, years	68.1 (60.5–73.4)	63.9 (50.0–70.0)	67.5 (60.0–73.0)	72.2 (66.0–75.2)	<0.001	69.8 (62.8–74.0)	65.1 (52.7–71.1)	66.3 (60.8–73.7)	73.0 (69.4–75.0)	<0.001
Male, %	50.5	47.8	46.3	56.5	0.31	45.8	35.7	43.2	54.8	0.11
**Primary renal disease, %**										
Glomerulonephritis	8.8	8.7	7.5	10.1	0.23	6.5	10.7	2.7	7.1	0.24
Diabetic nephropathy	15.9	6.5	19.4	18.8		15.0	7.1	18.9	16.7	
Hypertensive nephrosclerosis	18.7	17.4	17.9	20.3		26.2	21.4	27.0	28.6	
Other	26.9	26.1	26.8	27.6		28.9	21.5	29.8	33.3	
Unknown	29.7	41.3	28.4	23.2		23.4	39.3	21.6	14.3	
**Diabetes mellitus, %**	33.0	21.7	37.3	36.2		37.4	28.6	45.9	35.7	0.66
**Smoking, %**										
Never	62.6	63.0	67.2	58.0	0.79	65.4	60.7	73.0	61.9	0.89
Ex-smoker	23.1	23.9	19.4	26.1		20.6	21.4	16.2	23.8	
Current	14.3	13.0	13.4	15.9		14.0	17.9	10.8	14.3	
**History of cardiovascular disease, %**	28.0	26.1	28.4	29.0	0.94	37.4	35.7	35.1	40.5	0.66
**eGFR**										
CKD epi, ml/min/1.73m²	44.4 (31.6–78.1)	46.9 (34.6–93.3)	49.7 (27.4–73.7)	37.3 (24.6–60.9)	0.003	44.9 (32.0–74.5)	51.2 (34.7–96.1)	46.9 (34.7–78.3)	37.9 (21.8–64.1)	0.007
**CKD stage, %**										
CKD stage 1	8.8	21.7	6.0	2.9	0.005	15.0	35.7	10.8	4.8	0.006
CKD stage 2	27.5	15.2	38.8	24.6		23.4	7.1	32.4	26.2	
CKD stage 3	36.3	45.7	31.3	34.8		39.3	46.4	37.8	35.7	
CKD stage 4–5 (not on RRT)	27.5	17.4	23.9	37.7		22.4	10.7	18.9	33.3	
**Antropometric measures**										
Body mass index, kg/m^2^	29.2 (5.1)	27.6 (4.4)	28.7 (5.0)	30.7 (5.3)	0.001	30.1 (5.2)	28.6 (4.5)	30.0 (5.6)	31.3 (5.2)	0.04
Waist circumference, cm	103.8 (12.8)	100.1 (12.7)	102.5 (12.0)	107.4 (12.9)	0.003	106.9 (13.2)	103.3 (12.7)	106.1 (13.0)	109.9 (13.3)	0.04
**Blood pressure**										
Systolic blood pressure, mmHg	141.8 (17.5)	139.9 (19.9)	140.9 (14.7)	144.0 (18.3)	0.21	139.3 (17.3)	135.8 (19.1)	137.7 (14.1)	143.1 (18.4)	0.09
Diastolic blood pressure, mmHg	80.7 (10.6)	82.3 (10.0)	80.3 (11.0)	80.0 (10.7)	0.31	79.1 (11.1)	80.8 (9.7)	76.6 (10.1	80.1 (12.6)	0.81
Pulse pressure, mmHg	61.2 (16.7)	57.6 (19.0)	61.0 (13.5)	63.7 (17.6)	0.05	60.2 (15.5)	55.0 (16.4)	61.2 (11.9)	62.9 (17.1)	0.04
**Lipids**										
LDL/HDL	2.6 (1.0)	2.6 (1.6)	2.5 (0.75)	2.6 (0.66)	0.84	2.5 (1.2)	2.6 (1.9)	2.4 (0.82)	2.6 (0.68)	0.94
Tryglycerides, mg/dl	134.0 (101.0–191.3)	125.0 (87.0–170.0)	147.0 (104.8–193.5)	143.0 (100.5–210.0)	0.03	130.0 (99.0–178.0)	105.5 (70.5–132.3)	150.0 (106.5–186.5)	132.5 (100.5–190.0)	0.04
Inflammatory factors*										
CRP, mg/l	2.0 (0.73–4.5)	1.8 (0.40–4.0)	2.2 (1.00–4.73)	2.0 (0.62–5.0)	0.94	1.0 (0.31–3.5)	0.67 (0.14–2.3)	2.0 (0.55–4.6)	0.95 (0.36–4.1)	0.31
IL-6, pg/ml	3.1 (2.1–4.5)	2.4 (1.8–3.9)	3.0 (2.2–4.2)	3.8 (2.4–5.7)	0.007	3.4 (2.2–5.0)	2.3 (1.8–3.6)	3.3 (2.4–5.0)	4.2 (2.7–6.5)	<0.001
TNF-α, pg/ml	1.9 (1.4–2.9)	1.6 (1.3–2.5)	1.8 (1.4–2.7)	2.2 (1.7–3.4)	0.01	2.0 (1.4–3.0)	1.6 (1.3–2.6)	1.9 (1.3–2.6)	2.1 (1.7–3.5)	0.02
Endothelium factors*										
ICAM-1, ng/ml	254.5 (205.5–328.4)	245.0 (208.0–308.0)	257.8 (207.0–338.0)	255.0 (202.0–331.1)	0.68	231.0 (191.0–316.0)	226.5 (200.3–268.5)	230.0 (186.0–328.5)	242.0 (188.3–319.5)	0.87
VCAM-1, ng/ml	842.3 (623.0–1148.3)	761.1 (595.0–958.8)	907.0 (703.8–1297.3)	912.0 (655.0–1289.0)	0.009	814.0 (623.0–1018.0)	678.5 (583.8–796.8)	869.0 (704.5–1063.0)	918.0 (688.5–1278.0)	0.003
**Medications, %**										
ACE and/or ARBs	62.6	71.7	67.2	52.2	0.07	64.5	64.3	64.9	64.3	0.99
Diuretics	47.3	26.1	52.2	56.5	0.003	51.4	25.0	62.2	59.1	0.009
Aspirin	16.5	10.9	11.9	24.6	0.07	18.7	10.7	18.9	23.8	0.18
Statin	35.7	28.3	34.3	42.0	0.13	35.5	32.1	35.1	38.1	0.61
Calcium channel blockers	59.3	54.3	58.3	63.8	0.59	58.9	53.6	62.2	59.9	0.67

NA = not applicable;

RRT = renal replacement therapy;

Results of continuous variables are presented as mean (standard deviation) or median (25^th^ and 75^th^ percentile).

### Cross sectional study

**Fig 1:**
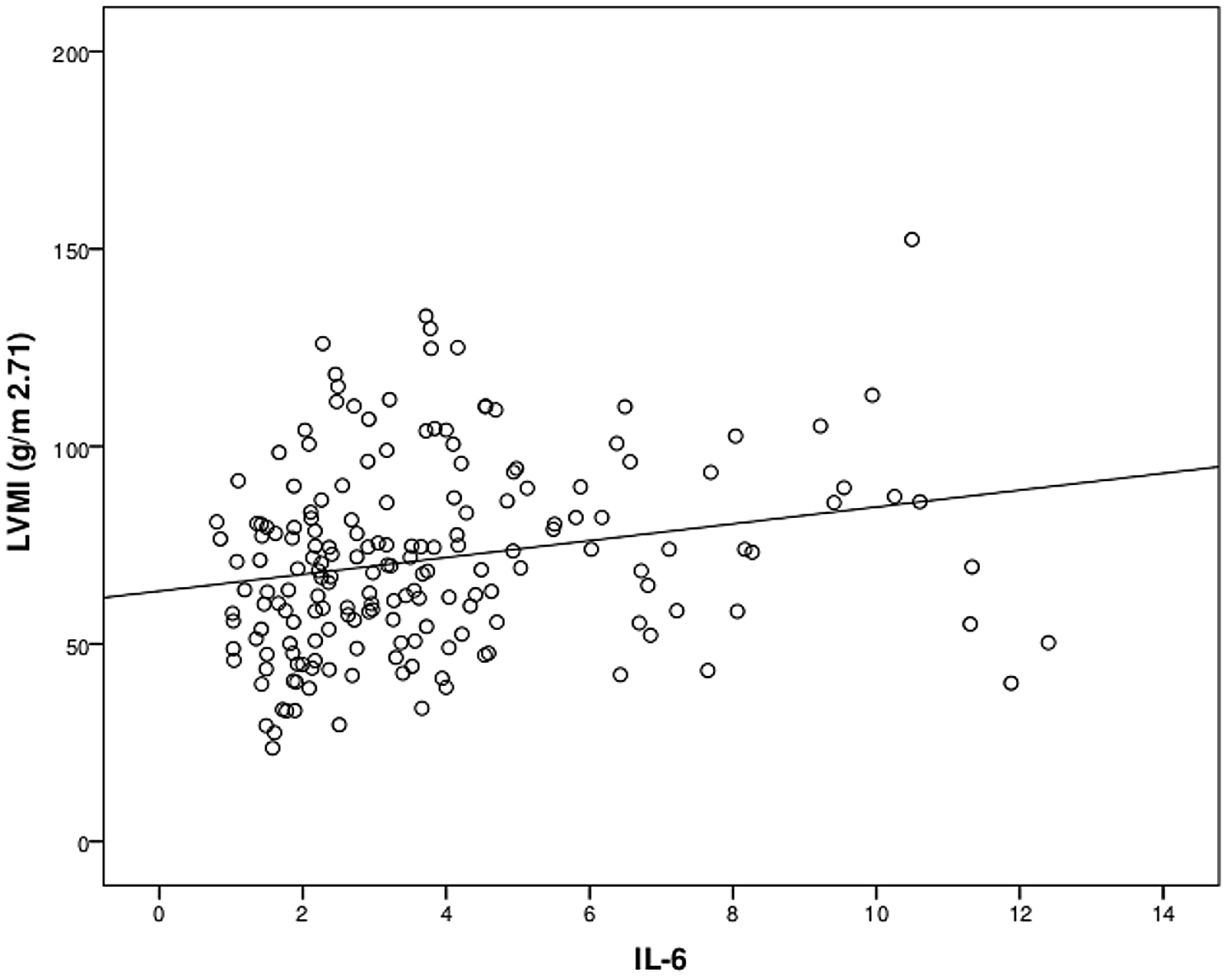
Correlation between IL-6 with left ventricular mass (g/m^2^·^71^) (n = 182).

**Fig 2:**
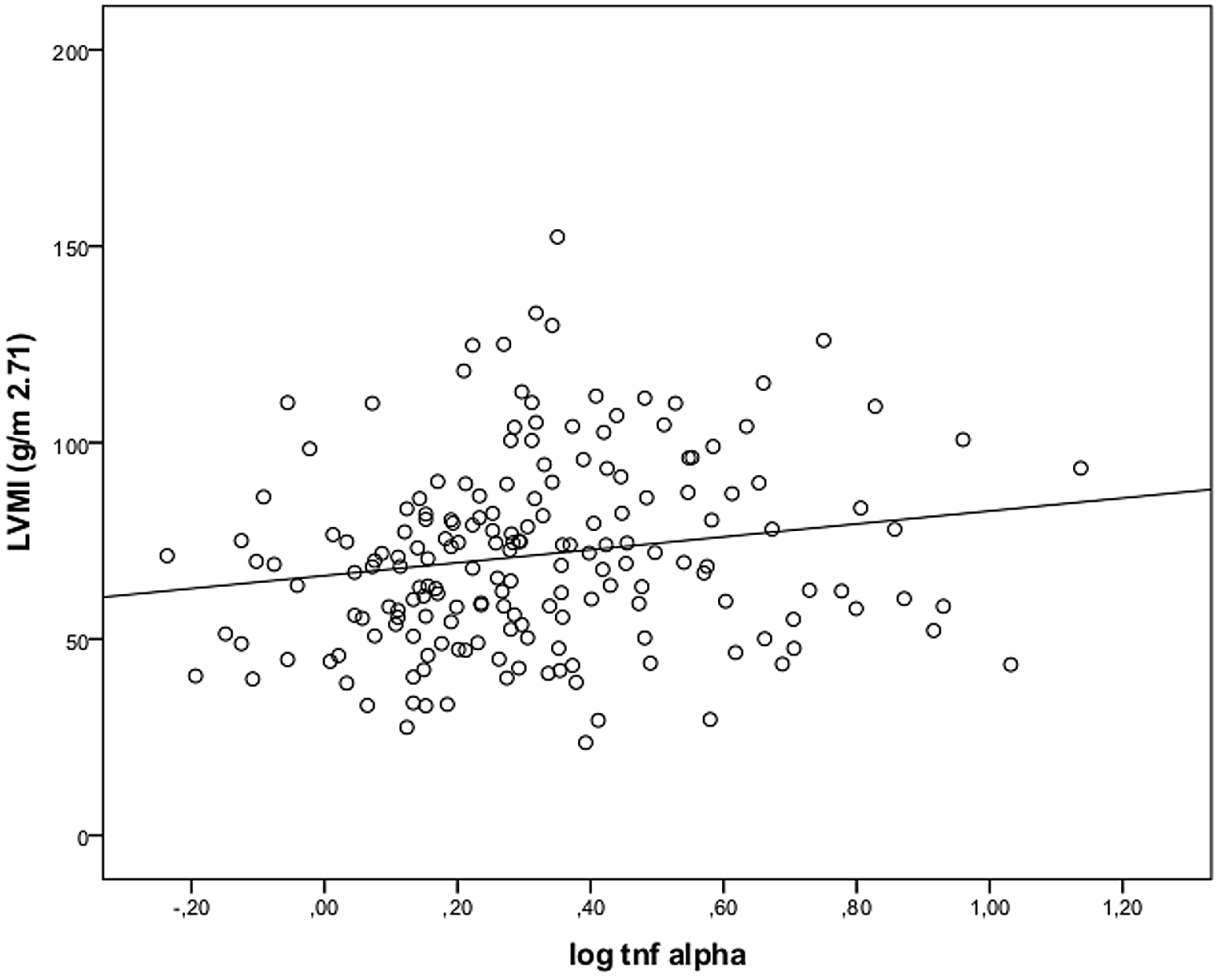
Correlation between log TNF-α with left ventricular mass (g/m^2^·^71^) (n = 182).

**Fig 3:**
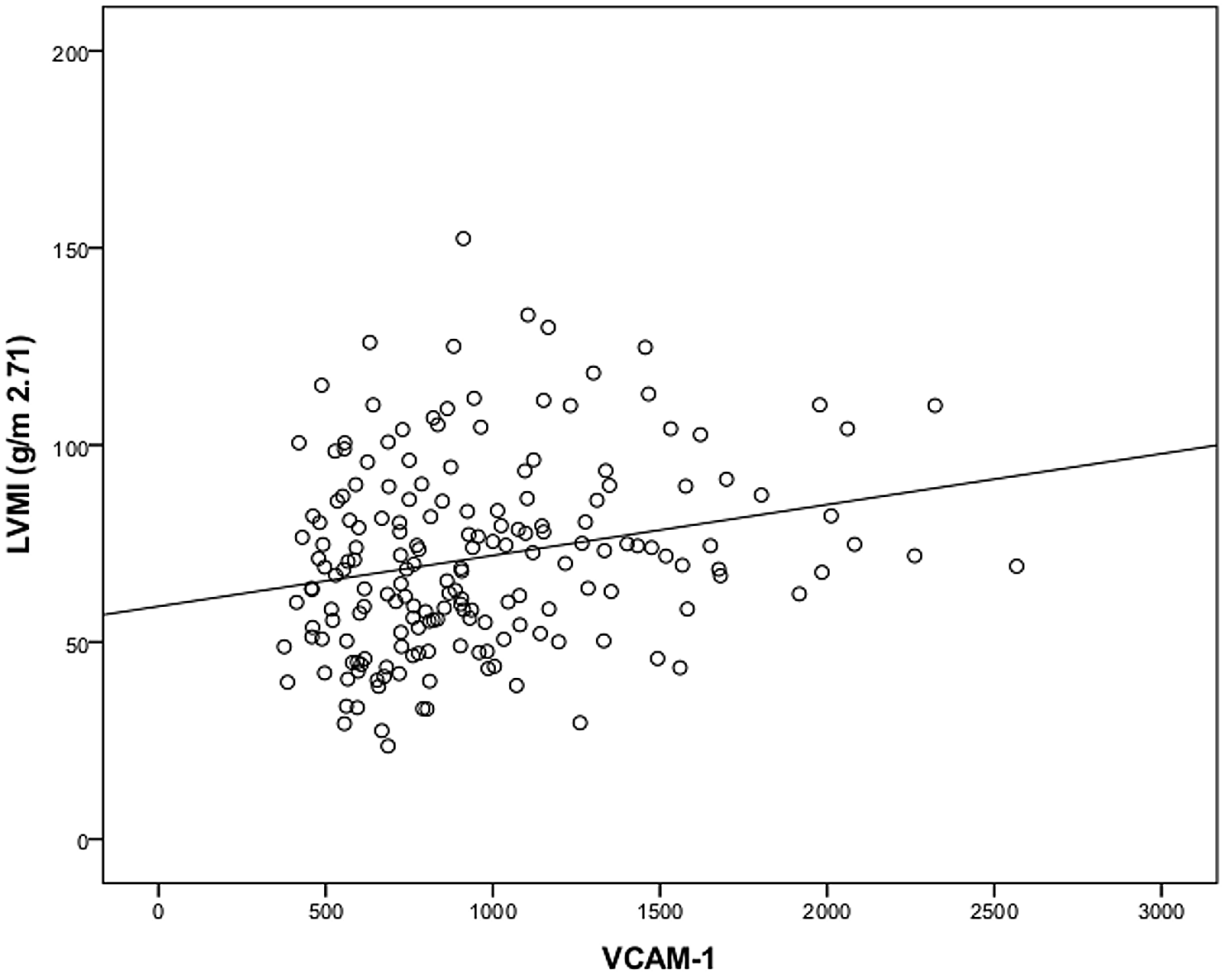
Correlation between VCAM-1 with left ventricular mass (g/m^2^·^71^) (n = 182).

At baseline, age, body mass index, waist circumference, tryglycerides, and pulse pressure increased with increasing LVMI (as categorized in tertiles, see Table 1) (p<0.05). Additionally, the proportion of patients using diuretics increased with increasing LVMI (p<0.05). Also, LVMI was strongly associated with CKD stage showing an inverse association with eGFR. Remarkably, IL6, TNFα, as well as VCAM-1 were directly associated with LVMI in bivariate analyses (Table 1, Figs 1, 2 and 3). Neither CRP nor I-CAM-1 were associated with LVMI.

Table 2 shows the unadjusted and adjusted β regression coefficients of the association between inflammation markers and endothelial dysfunction markers on the one hand and LVMI on the other hand. In model 2 we adjusted for potential confounders, whereas in model 3, we adjusted for the same confounders of model 2 plus eGFR (the putative mediator). This latter adjustment was made to examine to what extent the association between LVMI and endothelial dysfunction and inflammation markers could be explained by eGFR. After adjustment for confounders (model 2), a higher TNF-α, a higher inflammation score, and a higher VCAM-1 were all associated with a higher LVMI. The beta of log TNF-α substantially decreased from 15.3 in model 2 to 8.6 in model 3 becoming largely not-significant, suggesting that the association between TNF-α and with LVMI was partly mediated by eGFR (i.e. a higher TNF-α may result in a lower eGFR, which may increase LVMI). In comparison to the fully adjusted model (model 2), the beta’s of log CRP and their accompanying confidence intervals did hardly change when adjusting for less confounders.

**Table 2:** The association between both inflammation markers and endothelial dysfunction markers with left ventricular mass using linear regression analysis in cross sectional data.

	Left ventricular mass index, indexed to height (g/m^2^·^71^) (n = 182)		
	Model 1: Unadjusted	Model 2:Adjusted*	Model 3:Adjusted**
	Beta (95% confidence interval)		
Log CRP, mg/l	0.77 (-4.9; 6.4)	2.9 (-2.3; 8.0)	3.4 (-1.6; 8.5)
IL-6, pg/ml	**2.1 (0.72; 3.5)**	0.84 (-0.52; 2.2)	0.62 (-0.71; 2.0)
Log TNF-α, pg/ml	**16.5 (2.5; 30.3)**	**15.3 (2.5; 28.2)**	8.6 (-5.0; 22.2)
Inflammation score	**6.4 (2.5; 10.3)**	**4.6 (0.98; 8.2)**	**3.8 (0.12; 7.4)**
ICAM-1, ng/ml	0.02 (-0.02; 0.05)	0.03 (-0.008; 0.06)	0.03 (-0.003; 0.06)
VCAM-1, ng/ml	**0.01 (0.005; 0.02)**	**0.009 (0.002; 0.02)**	0.005 (-0.003; 0.01)

* Model 2: Adjusted for age, sex, primary renal disease plus confounders that additionally changed the beta ≥ 10%, i.e. smoking, ace or arb use, diuretic use, and statin use (log CRP); diuretic use (IL-6); smoking (log TNF-α); diuretic use (inflammation score);

** Model 3: adjusted for confounders of model 3 + eGFR. Significant results are in bold.

### Longitudinal study

Table 1 showed similar associations between the baseline characteristics and LVMI (as categorized in tertiles) in patients with baseline data (n = 182) and patients with follow-up data (n = 107) on LVM. An exception is the association between systolic blood pressure and LVMI, which was only statistically significant in patients with follow-up data.

Table 3 shows the longitudinal associations between the level of inflammation and endothelial dysfunction markers and LVMI over 3 years using linear mixed models. IL-6, the inflammation score and VCAM-1 were significantly associated with LVMI after adjustment for potential confounders (model 2). Remarkably, the beta’s of these significant associations hardly changed after additional adjustment of eGFR (model 3). implying that these longitudinal associations were largely independent of ongoing changes in the eGFR. In comparison to the fully adjusted model (model 2), the beta’s of log CRP and their accompanying confidence intervals did hardly change when adjusting for less confounders.

**Table 3:** The association between inflammation markers and endothelial dysfunction markers and left ventricular mass per year using linear mixed modelling in longitudinal data.

	Left ventricular mass index, indexed to height (g/m^2^·^71^),(n = 107)		
	Model 1: Unadjusted	Model 2: Adjusted*	Model 3: Adjusted**
	Beta (95% confidence interval)		
Log CRP, mg/l	0.22 (-0.23; 2.7)	-0.55 (-3.0; 1.9)	-0.57 (-3.0; 1.9)
IL-6, pg/ml	**3.5 (1.9; 5.2)**	**1.9 (0.36; 3.5)**	**1.8 (0.28; 3.4)**
Log TNF-α, pg/ml	13.8 (-0.06; 27.7)	6.5 (-6.1; 9.0)	5.6 (-6.6; 17.8)
Inflammation score	**9.6 (5.1; 14.1)**	**5.0 (0.72; 9.4)**	**5.2 (0.92; 9.5)**
ICAM-1 ng/ml	0.035 (-0.01; 0.08)	0.04 (-0.0003; 0.08)	**0.04 (0.005; 0.09)**
VCAM-1, ng/ml	**0.018 (0.0087; 0.027)**	**0.01 (0.005; 0.02)**	**0.01 (0.003; 0.02)**

* Model 2: Adjusted for age, sex, primary renal disease plus those confounders that additionally changed the beta ≥ 10%, i.e. smoking, ace or arb use, diuretic use, and statin use (log CRP); diuretic use (IL-6); smoking (log TNF-α); and diuretic use (inflammation score);

** Model 3: adjusted for confounders of model 3 + eGFR. Significant results are in bold.

## Discussion

Within a longitudinal study we for the first time show that inflammation as measured by IL-6 and a biomarkers-based inflammation score and endothelial dysfunction as measured by VCAM-1, is associated with LVMI worsening over time in patients across the whole spectrum of pre-dialysis CKD.

Inflammation and endothelial dysfunction play a central role in the atherosclerotic process [33, 34] and are considered of central importance in cardiovascular disease in CKD patients [14, 35]. Well beyond pressure and volume overload and comorbidities including obesity, infection and other environmental risk factors, CKD per se triggers a complex series of pro-inflammatory events engendering vascular damage and myocardial disorders [36–39]. In a large series of patients with untreated, uncomplicated essential hypertension and renal function decline, inflammation and endothelial dysfunction, an early step of the atherosclerosis process, per se contributed importantly to explain the variability in LVMI in these patients [40]. These observations in human disease are paralleled by experimental data in the rat where—independent of alterations in blood pressure, volume overload, plasma renin and angiotensin II and BNP- ablation of the 50% of functioning renal mass (a model of mild renal dysfunction) initiates myocardial gene responses involving a major inflammatory pathway conducive to TGF-β activation and fibrosis [41]. LVH and endothelial dysfunction are strictly inter-related phenomena and hallmarks of the response to salt load in the SHR [16] and in salt-sensitive forms of human hypertension [17]. Furthermore, it is well established that inflammation per se plays a role in the generation and the progression of myocardial hypertrophy to chronic heart failure [5].

In this study we for the first time show that IL-6 levels, a major cytokine which was associated to LVH in a study in dialysis patients based on genetic marker of this pro-inflammatory molecule [42], as well as the inflammation score, go along with longitudinal changes in LVMI. The relevance of the inflammatory pathway to explain the evolution of LVM over-time in CKD was corroborated by cross-sectional and longitudinal associations of an inflammation score based on CRP, IL-6 and TNF-α with LVMI. Inflammation markers composing this score at least in part reflect different facets of the inflammation process and may therefore have a complementary role in detecting inflammation. In a previous study by Zoccali et al. (2006) the combination of such markers (CRP, IL-6 and TNF-α) into a single score better predicted mortality than single markers in dialysis patients [30]. We did however not assess the prognostic value of the inflammation score in comparison to each inflammation marker alone, but rather performed an etiological study focusing on the causal association between the inflammation score and LVMI in pre dialysis CKD patients [43].

Among endothelial function markers we found that VCAM-1, but not ICAM-1, was associated with LVMI. VCAM-1 seems to have a more crucial role than ICAM-1 in mechanisms leading to increased LVMI, i.e. atherosclerosis and hypertension. Experimental data show that although expression of both VCAM-1 and ICAM-1 is upregulated in atherosclerotic lesions, VCAM-1, but not ICAM-1, plays a dominant role in the initiation process of atherosclerosis, since VCAM-1 is found in inflammatory regions predisposed to atherosclerotic lesions, while low levels of ICAM-1 are found in normal arterial segments expressed by normal endothelial cells [44]. Moreover, VCAM-1 but not ICAM-1 also associates with LVM in patients with uncomplicated essential hypertension [45], and in patients with hypertensive concentric LVH [46]. Therefore, VCAM-1 seems to have a more crucial role than ICAM-1 in the two major mechanisms leading to increased LVMI, i.e. atherosclerosis and hypertension.

### Strengths and limitations

A main strength of this study was the availability of longitudinal data on CRP and on LVMI which was assessed annually for a period of three years. To our knowledge, longitudinal studies examining the association between inflammation markers and endothelial dysfunction markers with LVMI in non-dialysis CKD patients are lacking. There are only few prospective studies on the association of such markers with all-cause mortality, cardiovascular mortality and morbidity in hemodialysis patients [12–15]. Another strength of this study is the inclusion of consecutive patients from all CKD stages in a prospective approach.

Our study also has some limitations. First, albuminuria was not included in the definition of CKD, because at the time of including patients for this study (in 2004 and 2005), we used the definition of CKD based on the guidelines of the Kidney Disease Outcomes Quality Initiative as published in 2002. Second, our study design is purely observational. Although we were able to adjust for many potential confounders, residual confounding remains possible because we could not adjust for confounders that were not measured or were unknown. Third, again due to observational nature of our findings, causality cannot be inferred from our data. However, in the quest for causality longitudinal studies rank higher than cross-sectional studies in the ladder of evidence [47] and as such it adds more solid support than previous cross-sectional studies that inflammation and endothelial dysfunction play a role in LVH in CKD patients. Finally, due to the smaller sample size especially in the longitudinal analyses a degree of caution is required in interpreting the results.

## Conclusion

This is the first longitudinal study in predialysis CKD patients on mechanisms of increased LVMI involving inflammation markers and endothelial dysfunction markers. In these patients, both IL-6 and an inflammation score based on CRP, IL-6 and TNF-α, as well as VCAM-1 were coherently associated with LVMI.

## Supporting Information

S1 File Dataset on patients with chronic kidney disease. (SAV)
